# Energy reserves mobilization: Strategies of three decapod species

**DOI:** 10.1371/journal.pone.0184060

**Published:** 2017-09-08

**Authors:** Hernán Javier Sacristán, Yamila Eliana Rodríguez, Nair De los Angeles Pereira, Laura Susana López Greco, Gustavo Alejandro Lovrich, Analía Verónica Fernández Gimenez

**Affiliations:** 1 Centro Austral de Investigaciones Científicas, CONICET, Houssay, Ushuaia, Argentina; 2 Instituto de Investigaciones Marinas y Costeras, Universidad Nacional de Mar del Plata, CONICET, FCEyN, Funes, Mar del Plata, Argentina; 3 Universidad de Buenos Aires, Facultad de Ciencias Exactas y Naturales, Departamento de Biodiversidad y Biología Experimental, Laboratorio de Biología de la Reproducción y el Crecimiento de Crustáceos, Ciudad Universitaria, Buenos Aires, Argentina; 4 CONICET-Universidad de Buenos Aires, Instituto de Biodiversidad y Biología Experimental y Aplicada (IBBEA), Ciudad Universitaria, Buenos Aires, Argentina; University of Southern California, UNITED STATES

## Abstract

In food deprivation assays, several different responses have been observed in crustaceans. However, studying energy reserves utilization among more than one species during the same starvation period has not yet been performed, particularly to discern whether the responses are due to intrinsic and/or environmental factors. We hypothesize that decapod species with similar feeding habits have the same strategies in the use of energetic reserves during starvation, even though they inhabit different environments. The aim of this study was to compare the energy reserves mobilization of three decapods species (*Cherax quadricarinatus*, *Palaemon argentinus* and *Munida gregaria*) with similar feeding habits, exposed to similar food deprivation conditions. The crayfish, shrimp and squat-lobster were experimentally kept at continuous feeding or continuous starvation throughout 15 days. Every 3^rd^ day, the midgut gland index (MGI), and the glycogen, lipid and protein contents were measured in the midgut gland (MG) and pleon muscle. *Palaemon argentinus* mobilized more reserves during starvation, followed by *C*. *quadricarinatus*, and the last *M*. *gregaria*. The starved shrimps presented low MGI, whereas MG showed a reduction in glycogen (from day 6 to 15), lipid (from day 3 to 15), and protein levels (at day 9 and 15) while in their muscle, lipid reserves decreased at days 3 and 6. In *C*. *quadricarinatus*, the most affected parameters in the MG were MGI, glycogen (from day 6 to 15), and lipids (at day 12 and 15). In the MG of *M*. *gregaria* only the glycogen was reduced during fasting from 3 to 15 days. Even though the three studied species have similar feeding habitats, we found that their energetic profile utilization is different and it could be explained by the habitat, life span, temperature, organ/tissue, and metabolism of the species. Our results may be useful to understand the several different responses of crustaceans during starvation.

## Introduction

In their natural habitats, crustaceans have to overcome and tolerate the scarcity or the total absence of food for short or long periods for different reasons: molting; seasonal environmental changes, along with community structure changes; or pollution [1, 2]. Physiological responses to starvation are further interpreted through ecological modulators. In natural habitats, for instance, it is important to understand physiological responses to fasting, as they could indicate whether organisms had reduced their feeding due to food availability (quantity and quality), and/or the cessation of feeding to reduce a predation risk. Therefore, measures of starvation are important for assessing the state of a population [3]. During the intermoult period of crustaceans, starvation is responsible for the re-allocation of energy resources for tissue maintenance, and survival, regardless of metabolic costs [4]. Induced experimental starvation can reveal which macromolecules (glycogen, lipid and protein) are used, and in what sequence the different energy compartments are depleted. In this context, the midgut gland and the muscle of crustaceans are key body parts because they hold the greatest amount of energy reserves [5], which can be mobilized during non-feeding periods.

In crustaceans, several different responses occur during food deprivation assays, such as dissimilar sequence of glycogen, lipid and protein mobilization, which drop or increase enzyme activities. This is the result of the vast diversity of environments that they inhabit and their long evolutionary history [5]. Other factors that contribute to the observed diversity of interspecific variability of energetic reserves mobilization are the dissimilar biochemical methods and distinct experimental starvation periods utilized by the different authors [6]. However, both conjectures, diversity of environments and/or different experimental protocols, have not yet been demonstrated. Therefore, although there are numerous studies dealing with nutrition of decapod crustaceans and several comparisons were made across species, none has previously compared the effect of starvation on different species with the same methodology. This type of study can compare species from different habitats, and with different phylogeny, life spans, life cycle, feeding habits, etc. In addition, it might also be possible to find a relationship between different species and their physiological features using the same experimental design and biochemical analysis. We hypothesize that, as the three decapod species present similar feeding habits, their strategies in the use of energetic reserves will be similar during starvation, even though they inhabit different environments.

The studied decapod species were *Cherax quadricarinatus*, *Palaemonetes argentinus* and *Munida gregaria*, which belong to the same suborder Pleocyemata, and different Infraorders: Astacidea, Caridea, and Anomura, respectively. These species are good biological models because, among other characteristics, they are phylogenetically dissimilar, they can be maintained under laboratory conditions, can accept a commercial diet, are easily caught in nature, and have starvation resistance [7–11].

Considering the phylogenetic relationships among the infraorders within the decapod Reptantia clade, the estimated divergence time for Astacidea, Caridea, and Anomura linages are 278, 263, and 309 million years ago, respectively [12]. We understand that interspecific comparisons must take into account the phylogenetic relationships among species due to their lack of statistical independence owing to a shared ancestry [13, 14]. However, our three-studied decapod species are evolutionarily distant; therefore, from an evolutionary point of view we considered them as independent units for the purpose of the present study.

In general, the three studied species present similar feeding habits, as omnivores and deposit feeders. As omnivores, they feed on benthic invertebrates and algae, and as deposit feeders, they ingest detritus. Specifically, the redclaw crayfish, *Cherax quadricarinatus* (Decapoda, Parastacidae), is a freshwater crustacean, native from northern Australia and southeast Papua New Guinea. In natural ecosystems, crayfishes have polytrophic feeding habits and have been described as predators, omnivores and/or detritivores, consuming a variety of macrophytes, benthic invertebrates, algae and detritus [15] and references therein. The shrimp *Palaemon argentinus* (Decapoda, Caridea), inhabits shallow lakes and streams of South America (in Argentina, Paraguay, Uruguay and southern Brazil) [16, 17]. *Palaemon argentinus* is omnivorous and detritivorous, and consumes algae, benthic macroinvertebrates and zooplankton [18]. The squat-lobster *Munida gregaria* (Decapoda, Anomura), is distributed on the continental shelves off southern South America, New Zealand and southern Australia [19]. This species is an omnivorous generalist and has two different and simultaneous feeding habits: as omnivore it feeds on crustaceans, polychaets and macroalgae, and as a deposit feeder on particulate organic matter [20].

This study was aimed to compare the physiological responses of *Cherax quadricarinatus*, *Palaemon argentinus* and *Munida gregaria* exposed to similar food deprivation conditions. Specifically, analytical biochemical parameters were determined through a midgut gland index and energy reserves quantification. This information may be useful to understand the different responses of crustaceans during food deprivation conditions.

## Materials and methods

### Animals

Redclaw crayfish were hatched from a reproductive female stock supplied by Centro Nacional de Desarrollo Acuícola (CENADAC), Corrientes, Argentina. Each ovigerous female (59.8 ± 3.2 g mean body weight) was maintained in an individual glass aquarium (60x40x30 cm, width x length x height). Each aquarium contained 30L of dechlorinated tap water and was continually aerated. The temperature was maintained at 27±1°C with ALTMAN water heaters (100W, precision ± 1°C), and the photoperiod cycle was 10 h light: 14 h dark. Each aquarium was provided with a PVC tube cave (10 cm of diameter and 25 cm long) [21]. Females were fed daily *ad libitum* with both *Elodea* sp. and commercial TetraColor granules TETRA^®^ (47.5% crude protein, 6.5% crude fat, 2.0% crude fiber, 6.0% moisture, 1.5% phosphorus, and 100 mg ascorbic acid/kg) according to Bugnot (2009) [22] and De Bock (2010) [23]. Juveniles became independent at stage 3 [24], and then, they were separated from their mothers. To reach the desired experimental weight, they were pooled and maintained under conditions described in previous studies [25, 26].

Shrimps *Palaemon argentinus* were obtained from the Nahuel Rucá pond, Buenos Aires, Argentina, considered as an unpolluted area [27]. The animals were collected with a hand net and transported to the laboratory. Shrimps were maintained in 70 L aquaria (60x35x25 cm width x length x height) with continually aerated freshwater. The temperature was maintained at 23±1°C by ALTMAN water heaters, and a photoperiod cycle of 10 h light: 14 h dark.

Squat-lobsters were captured in the Beagle Channel, Tierra del Fuego, Argentina, by trawling for 10 minutes using an epibenthic trawl with 1.8 m and 1.2 m of horizontal and vertical openings, and 10 mm of mesh size [28]. Animals were transported to the laboratory and kept in polypropylene aquaria tanks (65×50×30 cm, width x length x height) with a chilled seawater recirculation system at 6–8°C and a photoperiod cycle of 10 h light: 14 h dark. Water quality was maintained with mechanical (50 μm) and biological filters and a UV-sterilizer.

No specific permissions were required for the locations used to capture *Palaemon argentinus* and *Munida gregaria*, because these zones are not protected or private areas. The three studied species are not endangered or protected species.

### Experimental design

A total of eighty eight crayfish (5.26±1.46 g mean body weight in intermoult stage) were placed in individual glass containers (1500 mL) with 1400 mL of filtered water under continuous aeration. In order to maintain the temperature constant at 27±1°C, the containers were placed in aquaria (53x40x12 cm, width x length x height) with water heaters [9]. During the trial, the photoperiod cycle was set at 10 h light: 14 h dark and pH was set in a range of 7.3–8.4.

Five hundred twenty eight shrimps (0.16±0.04g mean body weight in intermoult stage) were placed in individual plastic containers (500 mL) with 400 mL of filtered water under continuous aeration. In order to maintain the temperature constant at 23±1°C, the containers were placed into aquaria (60x35x25 cm, width x length x height) with water heaters. During the trial, the photoperiod cycle was set at 10 h light: 14 h dark, and pH was set in a range of 7.9–8.9.

Eighty eight squat lobsters in intermoult stage (6.93±1.51 g mean body weight) were placed in individual plastic containers (700 mL) with seawater under continuous aeration. In order to maintain the temperature constant at 6±1°C, the containers were placed in aquaria tanks (25x37x10 cm, width x length x height) with chilled seawater. During the trial, the photoperiod cycle was set at 10 h light: 14 h dark, and pH was set in a range of 7.2–7.6.

The three species were fed daily *ad libitum* with TetraColor granules (TETRA^®^) and acclimated to the experimental conditions above described during 1 week before the experimental onset. Specifically, nutrient content in the diet is common for the three crustacean species; therefore, food is not a source of variability. The first day of the assay (time 0 (T0), initial control), 8 crayfish, 48 shrimps and 8 squat lobsters were weighed (precision 0.1 mg) and dissected, the midgut gland and pleon muscle extracted, and frozen at -80°C.

The remaining animals of *Cherax quadricarinatus* (n = 80), *Palaemon argentinus* (n = 480) and *Munida gregaria* (n = 80) were randomly and equitably distributed into two groups: half of each species on the fed group and the other half in the starved group. The fed group was daily fed *ad libitum* with TetraColor^®^ granules throughout the 15 days of the entire experimental period. The starved animals were not fed until day 15. During the experimental period, all containers were cleaned and water was renewed twice a week. Animals were sampled at every three days (T3, T6, T9, T12 and T15) when 8 crayfishes, 48 shrimps and 8 squat lobsters of the two groups, fed and starved, were weighed. Each midgut gland and pleon muscle was dissected and frozen at -80°C. Considering the small size of shrimps (and the subsequent small volume of the obtained organ and muscular tissue) 6 midgut glands and pleon muscles from the same experimental group and time were pooled (8 pools with 6 organs or tissues each), to ensure enough material for all analysis.

The wet midgut gland index (MGI) was calculated to assess the potential mobilization of metabolic reserves during the assay. This index was calculated according to Jones et al. (2000) [29] as MGI (%) = (midgut gland weight/whole body weight) x 100. At T0 and for each macromolecule (glycogen, lipid and protein), a ratio between the macromolecule in the MG and in the muscle pleon was also calculated.

### Energetic reserves of midgut gland and pleon muscle

Total lipids were extracted following Folch´s protocol [30]. Lipids were extracted by homogenizing pre-weighed samples of the midgut gland or muscle with a mixture of chloroform: methanol (2:1 v/v). The homogenate was filtered through a funnel with a filter paper to recover the liquid phase. Subsequently, liquid samples were washed with a NaCl solution (0.9%) to obtain two layers. Total lipids were determined by the sulfophospho-vanillin method [31]. This method consists of oxidizing cellular lipids to small fragments after a chemical digestion with hot concentrated sulfuric acid. After the addition of a solution of vanillin and phosphoric acid, a fuchsia complex was formed and its absorbance was read at 530 nm on a CINTRA 10e CBC spectrophotometer. The standard solution was prepared with commercial extra virgin olive oil (Cocinero, Molinos Río de la Plata S.A.).

Glycogen concentration in both the midgut gland and muscle was measured based on Lo´s protocol [32]. In a glass tube, 1 mL of 30% KOH saturated with Na_2_SO_4_ was added to the pre-weighed sample. Tubes with their screw cap were put in a boiling water bath for 1 h, and then cooled in ice. One milliliter of ethanol 96° was added to precipitate the glycogen. The samples were placed in ice for 30 min and then were centrifuged (ROLCO 2036) at 4500 rpm for 10 minutes. The glycogen precipitates were next dissolved in 1 mL of distilled water. An aliquot of 300 μL of the above glycogen solution was brought to a sample volume of 1 mL by the addition of distilled water, 1 mL of 8% phenol solution added, and 5 mL of H_2_SO_4_ was added rapidly. Subsequently, the tubes were allowed to stand for 10 minutes, then shaken and placed for 10–20 minutes in a water bath at 25–30°C, before readings were taken. The absorption spectrum was read at 490 nm and the standard solution was prepared with rabbit glycogen (Sigma G0885).

Finally, total soluble protein was evaluated with the Coomassie blue dye method according to Bradford´s protocol [33] using serum bovine albumin as the standard (Sigma A6003).

### Statistical analysis

All data are expressed as mean ± standard error. The statistical analyses were performed comparing the three treatments fed, starved and the initial control group (T0). All statistical analysis was performed with the same sample size in each studied species at each time (*Cherax quadricarinatus*: N = 8; *Palaemon argentinus*: N = 8 (8 pools of 6 animals/organ or tissues each); and *Munida gregaria*: N = 8). Data from the midgut gland index, glycogen and total lipids reserves, and soluble protein were analyzed by Generalized Linear Mixed Models (GLMMs) using InfoStat software (2015) [34]. The heterogeneous variance structure was modeled and the most parsimonious model was selected by comparison using the Akaike Information Criterion (AIC), and graphical inspection of their residue distribution. Post-hoc comparisons were performed using Fisher’s LSD test. For all analyses, residuals were analyzed for normal distribution via statistic of the Shapiro-Wilk test. The significance level was set at 0.05.

## Results

**Fig 1:**
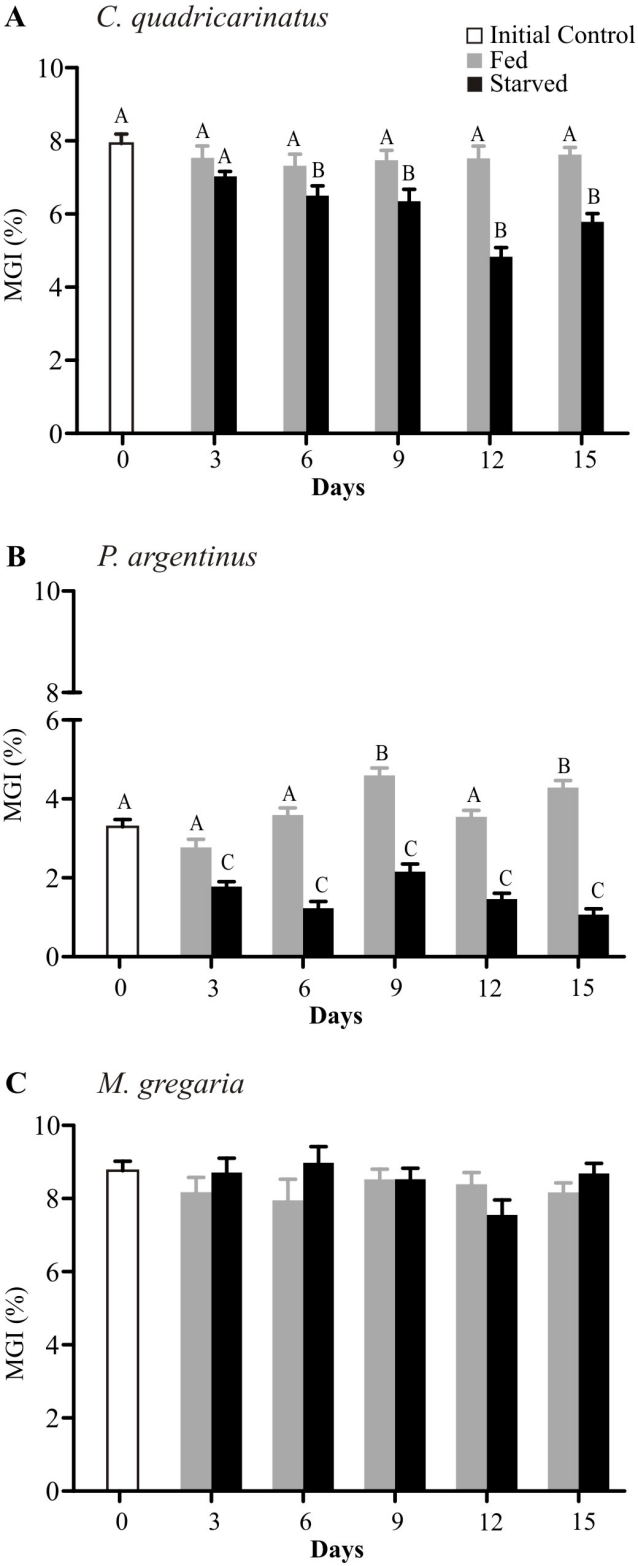
**Midgut gland index of *Cherax quadricarinatus* (A), *Palaemon argentinus* (B) and *Munida gregaria* (C) after starvation.** Different letters indicate statistical differences (p<0.05).

In starving condition, MGI changed in the crayfish and shrimp (Fig 1). From T6 to T15, starved crayfish had lower MGI values than fed animals and the initial control (Fig 1A). In starved shrimps, the MGI had lower values than T0 and feed animals at all sampling times (Fig 1B). On the other hand, MGI remained unchanged in *Munida gregaria* between treatments during the whole experiment (Fig 1C).

**Fig 2:**
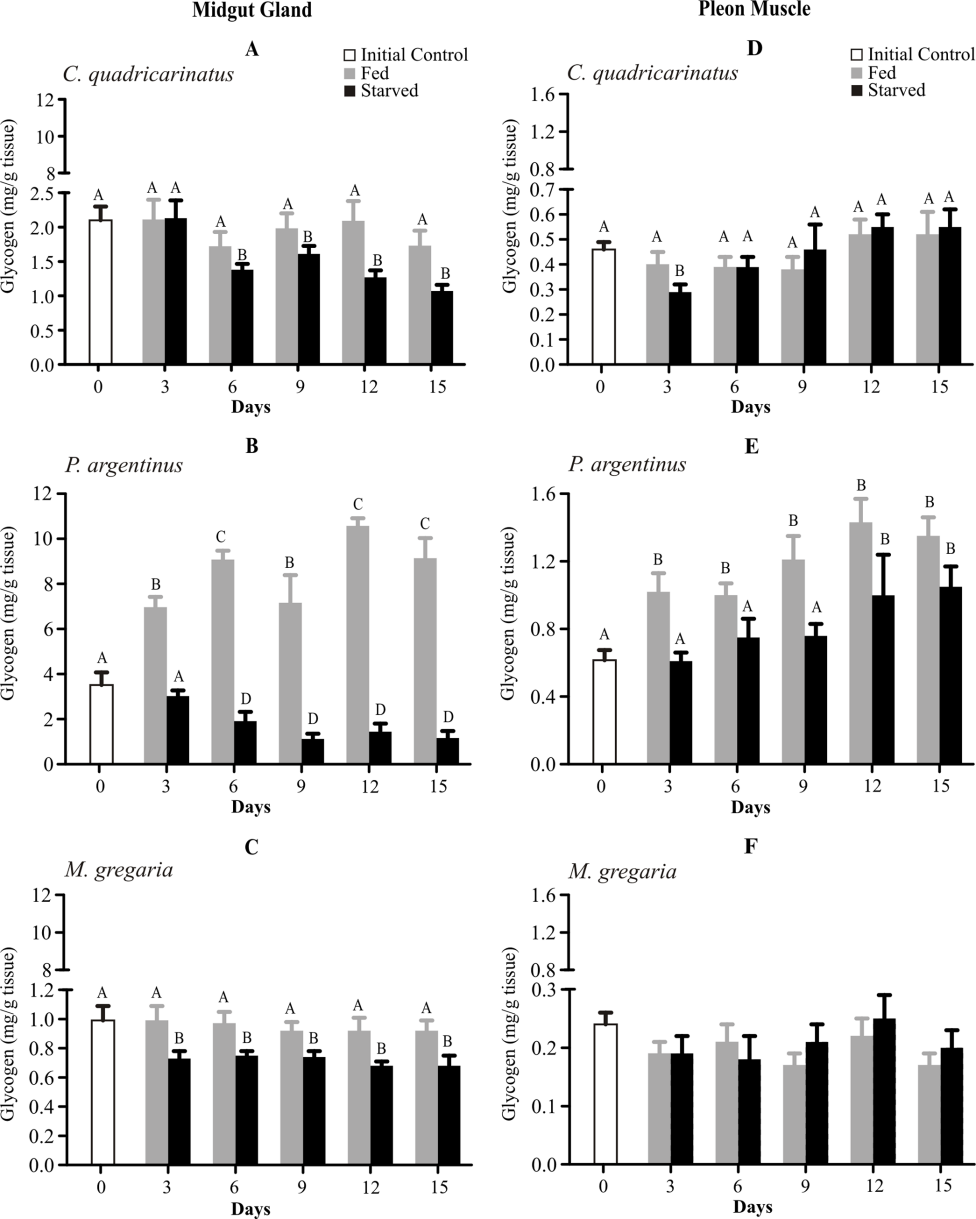
**Glycogen levels of midgut gland (left panel) and pleon muscle (right panel) of *Cherax quadricarinatus* (A, D), *Palaemon argentinus* (B, E) and *Munida gregaria* (C, F) after 15 days of starvation.** Different letters indicate statistical differences (p<0.05).

Crayfish started to consume midgut gland glycogen after day 6 of fasting (Fig 2A). Starved *Palaemon argentinus* reduced their glycogen reserves from T6 to T15 days compared to T0 and fed shrimps. In addition, in fed shrimps the MG glycogen concentration was higher than the control and throughout the experiment (Fig 2B). In starved *Munida gregaria* MG glycogen levels decreased earlier than in the other two species, after 3 days of starvation (Fig 2C). In the pleon muscle of both *Cherax quadricarinatus* and *Munida gregaria*, the glycogen reserves remained unchanged between treatments (Fig 2D and 2F). In *Palaemon argentinus* the glycogen concentration of the pleon muscle increased in both treatments: in fed animals at day 3, but later in starved animals, at day 12 (Fig 2E).

**Fig 3:**
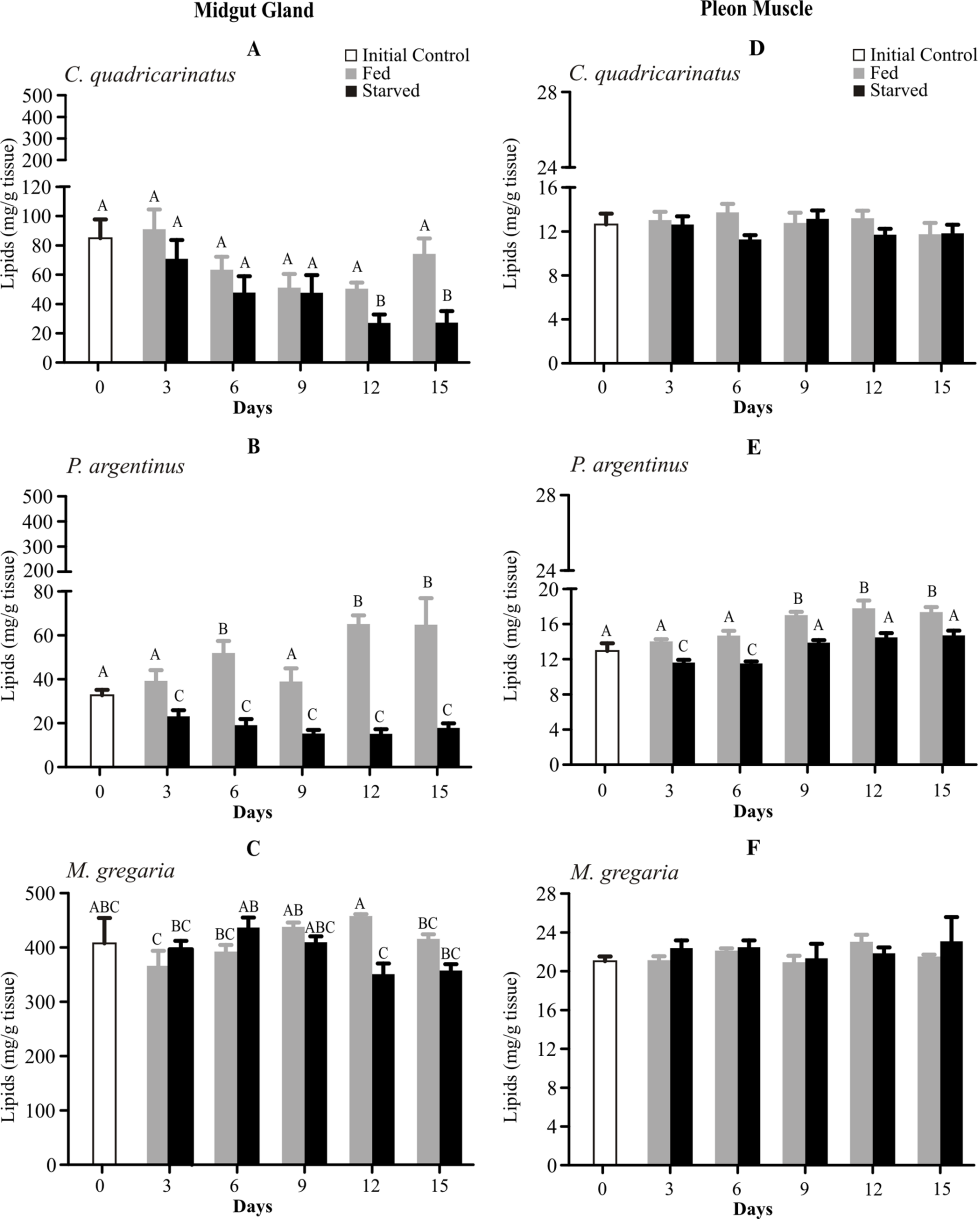
**Lipid levels of midgut gland (left panel) and pleon muscle (right panel) of *Cherax quadricarinatus* (A, D), *Palaemon argentinus* (B, E) and *Munida gregaria* (C, F) after 15 days of starvation.** Different letters indicate statistical differences (p<0.05).

Midgut gland lipids changed differently in the three species (Fig 3). In starved crayfish, MG lipid concentration was lower at T12 and T15 than in fed animals and the initial control (Fig 3A). Similarly, in starved squat lobsters MG lipids decreased only at T12 (Fig 3C). On the contrary, in the shrimp MG lipid concentration was lower in the starved condition during all the experiment, whereas fed animals showed an increment on lipid values at T6, T12 and T15 (Fig 3B).

Our results show that crayfish, shrimp and squat lobster did not use their muscle lipids during the 15 days of starving conditions (Fig 3). In particular, we found that just at T3 and T6, starved shrimps presented lower lipid levels than the fed ones. Also, fed animals accumulated lipid reserves from T9 (Fig 3E).

**Fig 4:**
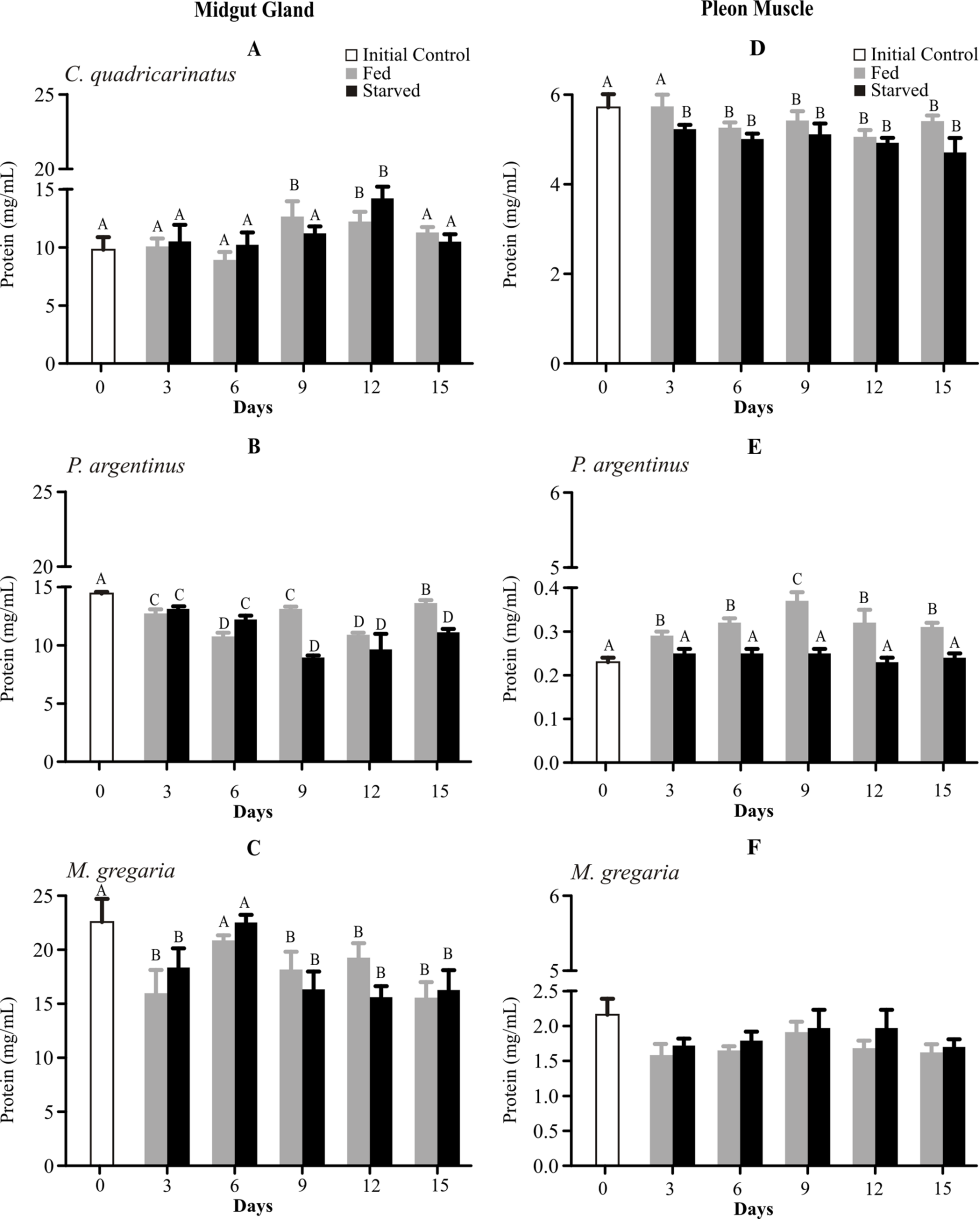
**Protein levels of midgut gland (left panel) and pleon muscle (right panel) of *Cherax quadricarinatus* (A, D), *Palaemon argentinus* (B, E) and *Munida gregaria* (C, F) after 15 days of starvation.** Different letters indicate statistical differences (p<0.05).

Protein levels of midgut gland remained unchanged in starved *Cherax quadricarinatus*, but decreased in *Palaemon argentinus* and *Munida gregaria* in both treatments (Fig 4). In fed *C*. *quadricarinatus*, proteins increased at T9 and T12, whereas in starved crayfishes proteins only increased at T12. Fed and starved shrimps showed lower protein values than initial controls at all times, and also were different at T6, T9 and T15 (Fig 4B). Similar results were observed in squat lobsters, where the midgut gland of fed and starved animals presented lower protein levels than T0, except at T6 (Fig 4C).

In starving condition, muscle protein levels remained unchanged in shrimps and squat lobsters, but in crayfish proteins were lower after 3 days (Fig 4). In fed *Cherax quadricarinatus* protein concentration was lower than T0 after 6 days (Fig 4D). On the other hand, during the whole experimental time, protein levels were higher in fed than in starved shrimps and T0 (Fig 4E).

At T0 the midgut gland had higher levels of all macromolecules than the muscle. In *Cherax quadricarinatus*, *Palaemon argentinus* and *Munida gregaria* respectively, the ratio MG:muscle for each macromolecule were: 4.5, 5.7 and 4 for glycogen; 6.7, 2.5 and 19.4 for lipid; and 1.7, 62.8 and 10 for protein.

## Discussion

Our results demonstrate that the three crustacean species present dissimilar physiological profiles and strategies in the utilization of energetic reserves during food deprivation despite having similar feeding habits. Specifically, *Palaemon argentinus* was the species that mobilized more kind of reserves during the 15 days of starvation, followed by *Cherax quadricarinatus* and *Munida gregaria*.

The midgut gland of starved shrimps presented low MGI, and a reduction in glycogen, lipid and protein levels; while in their muscle, lipid reserves decreased at days 3 and 6. In *Cherax quadricarinatus*, the most affected parameters in MG were MGI, glycogen and lipids. In the midgut gland of *Munida gregaria* only the glycogen concentration was reduced during fasting (Table 1).

**Table 1:** Summarized results of differences in the use of energy reserves of *Cherax quadricarinatus*, *Palaemon argentinus* and *Munida gregaria* after starvation.

	Parameter	*C*. *quadricarinatus*	*P*. *argentinus*	*M*. *gregaria*
**Midgut gland**	MGI	T6 –T15	T3 –T15	unchanged
	Glycogen	T6 –T15	T6 –T15	T3 –T15
	Lipid	T12, T15	T3 –T15	non utilized
	Protein	non utilized	T9, T15	non utilized
**Muscle**	Glycogen	T3	non utilized	non utilized
	Lipid	non utilized	T3, T6	non utilized
	Protein	non utilized	non utilized	non utilized

In general, as a response to starving the reduction in the midgut gland weight is due to macro-molecule changes (e.g. glycogen, lipid and protein). In *Cherax quadricarinatus* and *Palaemon argentinus*, such a drop in MGI is coincident with a decline in glycogen in crayfish, as well as glycogen and lipids in shrimp. On the contrary, during starvation, the midgut gland weight in *Munida gregaria* remained unchanged, despite its glycogen reserves depletion (Fig 2C).

In shrimps, lobsters and crayfish, glycogen stores are quickly depleted and likely converted to glucose to obtain energy e.g. [29, 35–40]. Under starvation, in *Munida gregaria* and *Palaemon argentinus*, the decrease of MG glycogen was fast and reached basal levels at day 3 (ca. 0.74 mg/g tissue) or day 6 (ca. 1.2 mg/g tissue), respectively. *Cherax quadricarinatus* showed a stronger starvation resistance, since during our 15 day starvation period the MG glycogen decreased, probably reaching its basal levels at day 15, thereafter remaining constant [9, 11, 41].

The present study suggests that the three studied crustaceans did not mobilize glycogen reserves from the tail muscle during the non-feeding period. The preservation of glycogen tail muscle may reflect its utility as a fuel in searching for food and/or tail-flip escape reaction. Nevertheless, in brachyuran crabs such as *Neohelice granulata* (Varunidae) or *Ocypode quadrata* (Ocypodidae) the muscle glycogen levels gradually decrease after 7 or 15 days of starvation, in order to maintain its energy requirements [42, 43].

The decline in MG lipid levels in *Cherax quadricarinatus* and *Palaemon argentinus* suggest that a lipolysis is taking place in the midgut gland as an energetic source. In the shrimp lipids probably have reached basal levels at day 9 (15.3 mg/g tissue) of starvation. Other species have a faster rate of lipid depletion, e.g. *Penaeus vannamei* (Penaeidae) reaches the lowest lipid levels after 4 days of starvation [38], and *Palaemon argentinus* uses 66% of the initial lipids during the first day of starvation [8]. Instead, in our work, *Palaemon argentinus* utilized ~ 30% of their lipid reserves throughout 3 starvation days. Lipid reduction, as a response to a different period of food deprivation (6–28 days), was also reported in other crustaceans such as the copepod *Acanthodiaptomus denticorni* (Diaptomidae); and the Penaeidae shrimps: *Penaeus vannamei*, *Penaeus duorarum*, *Penaeus semisulcatus*, *Penaeus monodon*, *Penaeus japonicus*, and *Penaeus esculentus* [35, 37, 38, 44–47].

There is no clear pattern that *Munida gregaria* utilizes its MG lipid reserves during the 15 days of food deprivation. However, this species has 5 and 13 times more lipids than *Cherax quadricarinatus* and *Palaemon argentinus*, respectively. The preservation of lipidic reserves could be related to the subantartic water temperature (5–10°C) in which *Munida gregaria* thrives, and hence preventing a possible cellular damage. Even though there are few studies about the relationship between unsaturated fatty acids and membrane fluidity in marine organisms [48], ectothermic animals increase membrane content of unsaturated fatty acids as a response to cold conditions to maintain its fluidity [48, 49].

Our results suggest that none of the three studied species utilize the energy from protein catabolism along the 15 days food deprivation period. During the experiment, soluble protein levels in midgut gland and muscle showed different fluctuation patterns, but in any case proteins were not spent. Starved prawns, shrimps or crabs can however, obtain energy through the catabolism of amino acids [37, 45, 50], but a prudent utilization of protein in short starvation periods may be an adaptive strategy to avoid the usage of high costly macromolecules, which could represent an energetic saving in case of prolonged periods without food [38]. For example, in the shrimp *Penaeus vannamei* soluble protein concentration remains relatively constant during 5 days of starvation [38]. Moreover, the lobster *Nephrops norvegicus* (Nephropidae) fasted through 12 weeks and 6 months, does not reduce protein levels in their tail muscle [51]. We hypothesize that the catabolism of protein and free amino acid pool might provoke the enzyme proteolysis and the lack of amino acid for the enzyme *de novo* synthesis, which could be essential in different metabolic processes for obtaining energy through the Krebs cycle, glycolysis, and fatty acid β-oxidation. Some authors suggest that in crustaceans the most important energy reserve compartments are the midgut gland and muscle [5]. However, our results in comparing the concentration of three macromolecules at T0 confirm that the midgut gland is the major organ of reserve storage, and that the tail muscle does not mobilize energetic resources due to the starvation stress in the same degree as the midgut gland.

Another interesting result of our research was that fed *Palaemon argentinus* increased lipid and glycogen levels in MG and lipid, glycogen and protein levels in pleon muscle, although the stored concentration is much higher in the midgut gland than in the muscle. This result confirms that this species has the capacity to accumulate more reserves, also illustrating the plasticity of this organ. The histological analysis of the midgut gland will be necessary to evaluate possible histopathological effects.

We are confident that our results are unbiased, even in the comparison of a permanent cultured species such as *Cherax quadricarinatus* with the other wild the species. Juveniles of crayfish were obtained in laboratory conditions from cultured individuals, with a regular feeding regime. *Palaemon argentinus* and *Munida gregaria* were captured from the wild, and therefore they might already have been subjected to starvation and could have a physiological advantage. *Cherax quadricarinatus* can recover from both short and long fasting periods. For example, in a regime of 4d of feeding and 4 d of starvation, energetic reserves remain unchanged [52]. After 50 days of starvation and 30 days of re-feeding, this crayfish recovers levels of glycogen, lipids, digestive enzyme activity, hepatopancreas structure, and regains its molting frequency [11]. Moreover, such a long fasting period can promote growth at low culture temperatures [53].

On the other hand, as the tested species thrive in different environments, i.e. marine or freshwater, they likely present different nutritional requirements. Wild animals used in the present study, *Munida gregaria* and *Palaemon argentinus*, can grab, manipulate, ingest, and metabolize the food they were offered at the beginning of the experiments. Several studies have demonstrated that wild crustaceans can be fed with TetraColor^®^. For example, the squat lobster fed exclusively with TetraColor^®^ can develop its ovary in a 4-month experiment [7] and successfully incubate embryos during their whole development [54]. *Palaemon argentinus* shrimp feeding on these commercial pellets can grow its midgut gland (Fig 1), and molt regularly (pers. obs.). Therefore, these results indicate that even though the diet may not be optimal for the three species, it is adequate for the animals to efficiently accomplish physiological processes with high energetic requirements, such as reproduction or molting. Furthermore, it is unknown whether feeding habits or food itself, could be under natural selection pressure, affecting directly the physiological fitness of these species.

Our results demonstrate that the species with the shortest life-span has mobilized more reserves during starvation: *Palaemon argentinus* (life-span ~1.5 yr) mobilized more reserves than *Cherax quadricarinatus* (life-span >3 yr), followed by *Munida gregaria* (life-span >5 yr) (Tables 1 and 2). Among decapods life-span varies considerably [55]. We did a literature review on the energetic mobilization of decapods during starvation in relation to their habitat and life-span (Table 2). The percentage of mobilization indicates the proportion of reserve reduction (considering the reserve concentration of fed animals as 100%) during the starvation period (Table 2). A comparison of the mobilization percentage among 21 decapod species shows a range from 28% to 99%. This mobilization is relatively low in species with longer life-span (>3 years) relativized to starvation days. Nevertheless, *Lithodes santolla*, *Munida gregaria* and *Penaeus esculentus* mobilize ~ 32% reserves during 60, 15 and 14 days of starving respectively; whereas, *Homarus americanus*, *Nephrops norvegicus*, *Procambarus clarkii*, *Cherax destructor*, and *Cherax quadricarinatus* mobilize more reserves (>60%) but during a longer period (102, 84, 210, 150, 154, and 80 days respectively) (Table 2). Therefore, we hypothesize that decapod species with longer life spans present lower proportion of reserve mobilization than species with short life-span, at similar starvation period. This pattern is also common to our studied species. Another approach shown in the literature is that marine decapods likely utilize glycogen reserves as an energy source during starvation independently of their life-span. By contrast, the starved freshwater decapods usually tend to utilize the three macromolecules: glycogen, lipids and protein (Table 2). In general, freshwater habitat is more variable than marine habitat with respect to temperature, current (lentic or lotic), nutrients (eutrophic or oligotrophic), and other factors. We further hypothesize that freshwater decapods present more plasticity to utilize more than one type of reserves due to variable habitat than marine decapods. Finally, the amount of different energetic reserves used by species could be related to the molt rate (as a proxy to the metabolic rate, given the numerous catabolic and anabolic processes involved in molting [56]; namely, at low molt rates the types of mobilized macromolecules will be few during starvation and vice versa (e.g *Lithodes santolla*, *Munida gregaria*, *Nephrops norvegicus*) (Table 2). This could be because a high molt rate would imply a high metabolism and, therefore, greater energy requirements would be needed during starvation to maintain homeostasis. In our three studied species molting frequency is variable: 40 d^-1^ [57], 45–50 d^-1^ (Sacristan unpublished data), and 2 yr^-1^ [28] in *Palaemon argentinus*, *Cherax quadricarinatus* and *Munida gregaria* respectively, and therefore this coincides with the type of reserve mobilized during starvation for the three species.

**Table 2:** Decapod preferential energy reserves mobilization according to habitat, life span, percentage of mobilization and starvation days in midgut gland and muscle.

						Reserve mobilization organ				
Reference number	Crustaceans	Infraorder/Suborder	Family	Habitat	Life-span(years)	Midgut gland	Muscle	Mobilization (%)	Starvation (days)	Reference
1	*Crangon crangon*	Caridea	Crangonidae	marine	3.3	glycogen	–	–	28	[58]
2	*Homarus americanus*	Astacidea	Nephropidae	marine	>31	glycogen	–	60	102	[59]
3	*Lithodes santolla*	Anomura	Lithodidae	marine	~14	glycogen	lipid	35	60	Sacristan et al unpublished
4	*Munida gregaria*	Anomura	Munididae	marine	>5	glycogen	non utilized	32	15	presente study
5	*Nephrops norvegicus*	Astacidea	Nephropidae	marine	15	glycogen	glycogen	87	210	[39]
6	*Ocypode quadrata*	Brachyura	Ocypodidae	marine	3	lipid	glycogen	53	15	[43]
7	*Penaeus vannamei*	Dendrobranchiata	Penaeidae	marine	1.5–2	glycogen and lipid	–	80 and 84	5	[38]
8	*Penaeus duorarum*	Dendrobranchiata	Penaeidae	marine	1.3–1.6	glycogen	–	–	–	[35]
9	*Penaeus esculentus*	Dendrobranchiata	Penaeidae	marine	2.4	–	protein and lipid	28 and 33	14	[45]
10	*Penaeus japonicus*	Dendrobranchiata	Penaeidae	marine	~1.3	glycogen	lipid	72	28	[37]
11	*Portunus pelagicus*	Brachyura	Portunidae	marine	3	glycogen	–	99	6	[60]
12	*Hemigrapsus nudus*	Brachyura	Varunidae	marine	–	protein	–	–	23	[61]
13	*Neohelice granulata*	Brachyura	Varunidae	brackish	3	glycogen	–	80	7	[42]
14	*Orconectes limosus*	Astacidea	Cambaridae	freshwater	>1.5	lipid	–	28	41	[62]
15	*Orconectes virilis*	Astacidea	Cambaridae	freshwater	>1.5	protein and carbohydrates	protein and carbohydrates	42 and 37	14	[63]
16	*Procambarus clarkii*	Astacidea	Cambaridae	freshwater	3.5–6.5	glycogen, lipid and protein	glycogen, lipid and protein	80, 80 and 89	150	[64]
17	*Procambarus zonangulus*	Astacidea	Cambaridae	freshwater	>1.5	glycogen, lipid and protein	glycogen, lipid and protein	87, 60 and 60	150	[64]
18	*Macrobrachium rosenbergii*	Caridea	Palaemonidae	freshwater	–	glycogen	–	74	4	[65]
19	*Palaemon argentinus*	Caridea	Palaemonidae	freshwater	1.3	glycogen, lipid and protein	lipid	66, 45 and 38	15	present study
20	*Cherax destructor*	Astacidea	Parastacidae	freshwater	>3	carbohydrates, lipid and protein	–	77, 98 and 61	154	[29]
21	*Cherax quadricarinatus*	Astacidea	Parastacidae	freshwater	>3	glycogen and lipid	non utilized	49 and 68; 72 and 95	15; 80	present study; [11]

**Fig 5:**
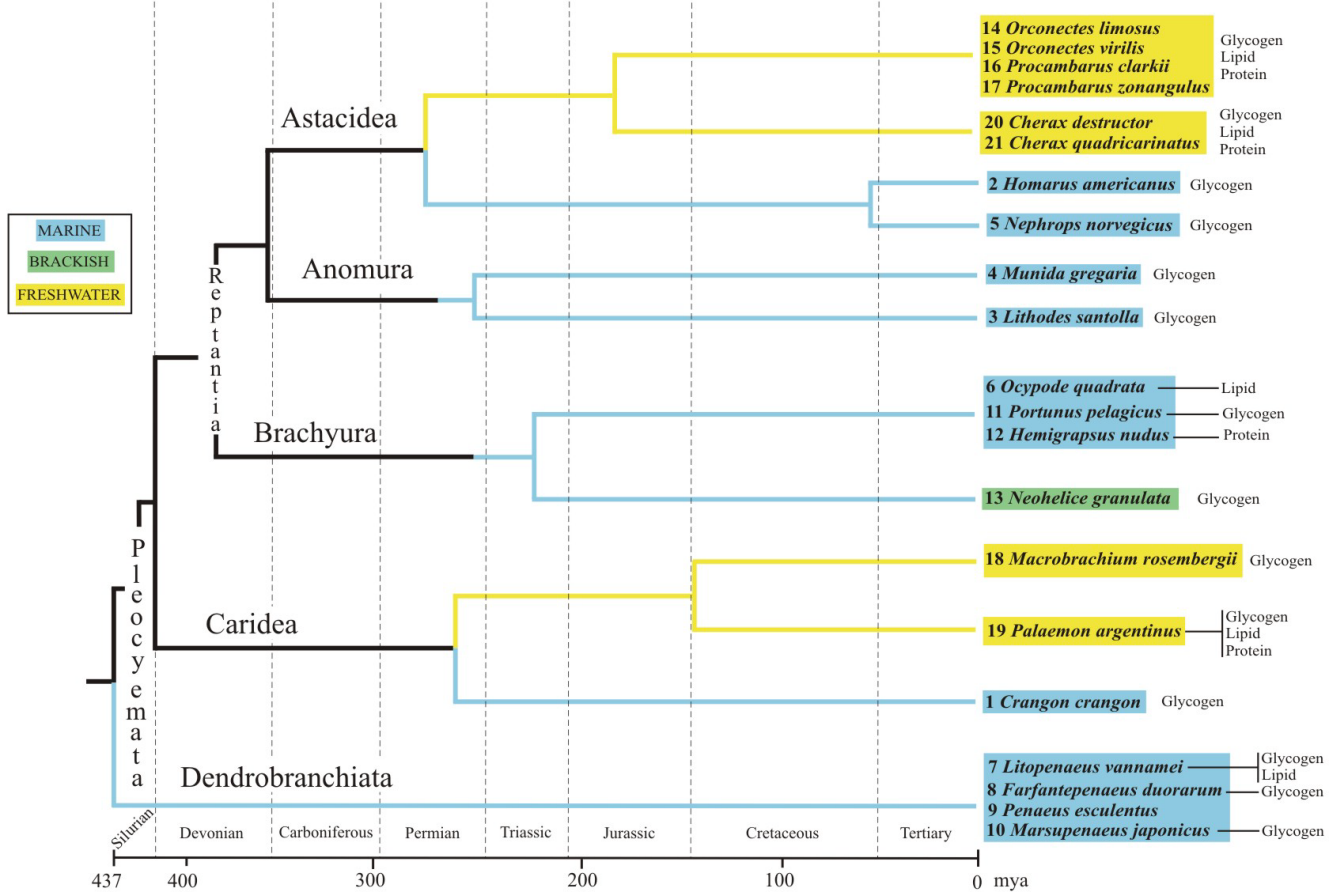
Energetic reserves mobilization of midgut gland, environments, and phylogenetic relationship among decapods crustaceans. Phylogenetic tree adapted from Porter et al. (2005) [12]. Numbers on left to the species names are the reference numbers of Table 2. Different color boxes (light blue, green and yellow) indicate the habitat of species.

Crustacean decapods display stronger relationships between the type of mobilized reserve and habitat, than phylogenetic distance among species (Fig 5). Specifically, infraorder Astacidea contains (short phylogenetic distance): astacids and nephropids, each one a freshwater or a marine lineage respectively. Freshwater crayfishes likely utilize the three kind of reserves as an energy source during starvation. Instead, marine clawed lobsters consume glycogen reserves independently of their phylogenetic relationship within the Astacidea. In the Caridea lineage, similar responses are observed between freshwater (Palaemonidae) and marine (Crangonidae) species (Fig 5). Moreover, marine species usually tend to utilize glycogen reserves during fasting periods, e.g. infraorders Anomura, Brachyura, and the suborder Dendrobranchiata (penaeid shrimps and their relatives) (Fig 5). Therefore, glycogen reserves consumption as a preferential energy source in the midgut gland could be considered as an ancestral character in decapods.

The sea realm is the ancestral environment of the Decapoda, with conquest of freshwater habitats being a later adaptation [66, 67]. In the freshwater prawn of genus *Macrobrachium*, and in lineages that split later, several adaptations associated with the transition to fresh water habitat or “freshwaterization” process were observed, such as reproductive adaptations, larval development and starvation resistance [68] [67] and references therein. In this sense, the ability to catabolize different compounds under short fasting periods may have emerged as an adaptive advantage to colonize more unstable environments such as freshwater. This capacity could have arisen more recently in the evolution of the organisms that inhabit freshwater habitats, and could have appeared more than once, since it can be observed in Caridea and Reptantia (Fig 5). Therefore, the physiological responses perceived the present study and previous works could indicate that freshwater decapods have acquired the ability to mobilize more than one kind of energetic reserve as part of the freshwaterization process. We hypothesize that in the evolutionary history of decapod species could have arisen a certain character that allowed to develop different mechanisms in response to starvation. Nevertheless, more comparative research between related species from different habitats and dissimilar phylogenetic distance are needed to test this hypothesis.

Considerable research has been focused on nutrition of decapod crustaceans and many comparisons have been made across species. This is the first study, which uses the same methodology and compares three decapod species with similar feeding habits living in different environments, and with patterns of energetic mobilization that are comparable across other decapod species. Nevertheless, for future works we propose the use of biochemical methods instead of proximal composition analyses for the study of decapod starvation response in order to test our hypothesis, and thus avoid the variability factor associated to the methodology employed. Specifically, reserve mobilization may be explained through intrinsic factors, such as life span and molt rate, as well as modulating environmental factors (e.g. temperature), habitat and phylogenetic relationships. Therefore, the present study shows that decapod crustaceans display a vast diversity of reserve mobilization strategies to deal with starvation, and suggests that these strategies are not related to the type of food. Finally, according to our results, the literature reviewed, and the hypothesis suggested above, presumably a variety of shared trends takes place in the physiological responses of decapod crustaceans during starvation. However, we could not confirm experimentally this assumption as it is necessary to include a number of species per habitat in the same study along with the use of an identical methodological approach.
